# Temperature-Promoted Giant Unilamellar Vesicle (GUV) Aggregation: A Way of Multicellular Formation

**DOI:** 10.3390/cimb45050242

**Published:** 2023-04-26

**Authors:** Xinmao Wang, Yangruizi Zhang, Maobin Xie, Zhibiao Wang, Hai Qiao

**Affiliations:** 1 State Key Laboratory of Ultrasound in Medicine and Engineering, College of Biomedical Engineering, Chongqing Medical University, Chongqing, 400016, China; 2 Chongqing Key Laboratory of Biomedical Engineering, Chongqing Medical University, Chongqing 400016, China

**Keywords:** giant unilamellar vesicles, aggregation, phospholipid, zeta potential, headgroup reorientation

## Abstract

The evolution of unicellular to multicellular life is considered to be an important step in the origin of life, and it is crucial to study the influence of environmental factors on this process through cell models in the laboratory. In this paper, we used giant unilamellar vesicles (GUVs) as a cell model to investigate the relationship between environmental temperature changes and the evolution of unicellular to multicellular life. The zeta potential of GUVs and the conformation of the headgroup of phospholipid molecules at different temperatures were examined using phase analysis light scattering (PALS) and attenuated total reflection-Fourier transform infrared spectroscopy (ATR-FTIR), respectively. In addition, the effect of increasing temperature on the aggregation of GUVs was further investigated in ionic solutions, and the possible mechanisms involved were explored. The results showed that increasing temperature reduced the repulsive forces between cells models and promoted their aggregation. This study could effectively contribute to our understanding of the evolution of primitive unicellular to multicellular life.

## 1. Introduction

One of the central events in the origin and evolution of life is the evolution of unicellular life into multicellular life [1,2,3]. Experimental studies have demonstrated that evolution to multicellular life can be achieved under the right physicochemical conditions [4,5,6]. Temperature, as the most fundamental physical condition in the environment, not only affects life activities, but it also affects the evolution of life [7,8,9], such as warming, which can promote the evolution of life and consequently help organisms to adapt to thermal variation [10]. However, there are no reports on the relationship between temperature changes and the evolution of unicellular to multicellular life. To better understand the effect of temperature change in the primitive environment on the evolution of unicellular life to multicellular life, it is necessary to conduct this study using cell models.

Several factors, including pH change [11,12], temperature change [13], ion concentration change [14], protein ligands [15,16], and DNA [17,18], can promote the aggregation of large unilamellar vesicles (LUVs). The aggregation degree of LUVs is generally reflected by dynamic light scattering (DLS) and electron microscopy (EM). Although DLS can indirectly represent the degree of overall vesicle aggregation through size discrepancy, it is difficult to observe their aggregation in solution via DLS directly. EM is ideal to directly observe vesicle aggregation, but it does not truly reflect the state of vesicles in the original solution due to its complicated sample preparation process, which alters the vesicle environment. Compared to LUVs, giant unilamellar vesicles (GUVs) could be observed directly in the original solution by inverted fluorescence microscopy or confocal laser scanning microscopy (CLSM). GUVs are close to cell size and are widely used as cell models or cell membrane models to study cell structure and function [19]. Several studies have shown that a number of factors could promote GUVs aggregation, including macromolecular interactions [20], pH changes [21], and ion concentration changes [22]. However, there are still no studies on the relationship between temperature and GUV aggregation.

Temperature, being one of the most critical factors in the environment, has a significant effect on vesicle aggregation. Previous studies have reported that the temperature could promote LUV aggregation in three main ways: (1) temperature increase could cause the headgroups of phospholipid molecules in vesicles to tilt to the bilayer plane, lowering the vesicle zeta potential and promoting vesicle aggregation [23]. (2) Temperature increase could promote the dehydration of the headgroups of phospholipid molecules in vesicles, decreasing the hydration repulsion between vesicles and facilitating vesicle aggregation [24,25]. (3) Temperature increase could promote the hydrolysis of phospholipid molecules in vesicles, and the hydrolysis product fatty acids can also contribute to vesicle aggregation [26]. Whether these mechanisms above are applicable to illustrate the promotion of GUVs aggregation by elevated temperature remains to be investigated.

Herein, GUVs were selected as the cell models in this study. The degree of aggregation of the cell models was firstly investigated in relation to temperature. Next, the mechanisms involved in this process were investigated by examining the zeta potential of GUVs and the conformation of the headgroup of phospholipid molecules. Finally, the effect of temperature change on the degree of aggregation of GUVs was researched in ionic solutions, and the potential mechanisms involved were explored. The results of this paper help to deepen our understanding of the evolution of primitive unicellularity into multicellularity.

## 2. Materials and Methods

### 2.1. GUVs Preparation

GUVs were prepared using 1,2-dimyristoyl-*sn*-glycero-3-phosphocholine (DMPC) (purity > 99%, 850345P, Avanti, Tonawanda, NY, USA) or 1,2-dipalmitoyl-*sn*-glycerol-3-phosphocholine (DPPC) (purity > 99%, 850355P, Avanti, Tonawanda, NY, USA) or 1,2-distearoyl-*sn*-glycero-3-phosphocholine (DSPC) (purity > 99%, 850365P, Avanti, Tonawanda, NY, USA) by electroformation according to the previous reports [27]. The preparation method of GUVs is as follows. First, 20 μL of phospholipid solution (10 mg/mL in chloroform) was coated on the conductive surface of two indium tin oxide glass plates (Zhuhai Kaivo Optoelectronic Technology Co., Ltd., Zhuhai, China). Then, after drying in a vacuum at 60 °C overnight, the two glass plates were assembled with their conductive side facing each other and separated by a 2 mm thick teflon frame to form a chamber that was sealed with vacuum silicon grease. At the next stage, the chamber was filled with RNase-Free water (CW0612M, Cwbio, Beijing, China). The glass plates were connected to a function generator (AFG31022, Tektronix, Tokyo, Japan), and the chamber temperature was maintained above the phospholipid phase transition temperature (DMPC: 45 °C; DPPC: 60 °C; DSPC: 65 °C). Finally, an alternating current of 2.5 V (the electric field strength in the chamber is 12.5 V/cm) at 10 Hz was applied for 4 h, during which GUVs formed within the chamber. 3,3′-Dioctadecyloxacarbocyanine perchlorate (DiO) (purity ≥ 98%, D4292, Sigma, St. Louis, MO, USA) or 1,1′-Dioctadecyl-3,3,3′,3′-tetramethylindo carbocyanine perchlorate (DiI) (purity ≥ 98%, 42,364, Sigma, St. Louis, MO, USA), a small molecular fluorescent dye (10 μM), was added to the phospholipid’s solution to observe the morphological and quantitative changes in GUVs.

GUV mixed solutions (mGUVs) were obtained by mixing equal volumes of DiO-GUVs solution and DiI-GUVs solution. GUVs in 1 mM NaCl solution were obtained by diluting normal saline 154-fold with the freshly prepared solution of GUVs. To avoid the influence of a high local concentration on the morphology of GUVs, normal saline was added slowly and mixed rapidly into the solution.

### 2.2. MLVs Preparation

Multilamellar vesicles’ (MLVs) preparation was through the thin film resolvation technique, as follows. First, 10 mg of DPPC phospholipids were dissolved in chloroform and stored overnight at 60 °C under a vacuum to deposit the phospholipid dry lipid film onto the glass vial walls. Additionally, then, the dried lipid film was dispersed into RNase-Free water using an ultrasonic cleaner (KQ2200, Kunshan Ultrasonic Instrument Co., Ltd., Kunshan, China) to prepare a turbid solution of 4 mg/mL. Samples were sonicated (56 W, 5 s interpulse interval) in an ice-filled ultrasonic water bath for 5 min (to minimize the effect of metal particles shed from the tip) via an ultrasonic probe (VCX150, SONICS, Newtown, CT, USA).

### 2.3. GUVs Solutions Incubation and Imaging

GUVs solution were incubated in the dark at temperatures of 4 °C, 25 °C, 37 °C, and 45 °C. Samples were taken on day 0, day 1, day 3, day 5, and day 7 of incubation, respectively. Before sampling, the homogenous solution was thoroughly mixed. Then, 10 μL of the GUVs solution was dropped into the glass bottom cell culture dish (801001, NEST, China), sealed with mineral oil (M5904, Sigma, USA), and allowed to settle for 3 h in the dark. The samples were viewed using an inverted fluorescence microscope (RVL-100-G, ECHO, San Diego, CA, USA) or a confocal laser scanning microscope (CLSM, A1R; Nikon, Tokyo, Japan), and the center of the GUVs solution and eight directions around it were photographed and recorded. Experiments were repeated three or more times and used for subsequent data analysis. Individual GUVs or aggregates were observed with a high-power lens and the Z-axis layer sweep recombination technique of CLSM (thickness is 0.5 μm). The excitation and emission wavelengths of DiO are 488 nm and 500–520 nm, and the excitation and emission wavelengths of DiI are 551 nm and 569 nm, respectively.

### 2.4. Zeta Potential Measurements

The zeta potential of GUVs was measured using phase analysis light scattering (PALS) method within the zeta potential analyzer system (NanoBrook Omni, Brookhaven, Holtsville, NY, USA). Before measurement, the electrode surface was mechanically cleaned by the electrode cleaning brush and deionized water to reduce errors. The effect of different electric field strengths (5.55 V/cm, 11.11 V/cm, 22.22 V/cm) on the zeta potential of GUVs and morphology of GUVs was first compared, and subsequent measurements were decided in 11.11 V/cm. The temperature gradually increased from 20 °C to 50 °C at 5 °C intervals with a stabilization time of 15 min after temperature adjustment. After that, each temperature node was measured for 15 cycles. The trial was repeated three or more times independently to determine its reproducibility.

### 2.5. ATR-FTIR

To scan the interferogram, RNase-Free water and DPPC-MLVs suspension dispersion droplets were separately placed using a micropipette, on an attenuated total reflection (ATR) crystal surface attached at an ATR accessory sample compartment as background and sample, respectively. The droplet temperature was regulated by a digital temperature controller unit. Infrared spectra were obtained using a Fourier transform infrared spectroscopy (FTIR) spectrometer (iS60, Nicolet, Waltham, MA, USA), operated with Spectrum software under ambient conditions at room temperature. Interferograms were collected for a wavenumber range from 3000 to 950 cm^−1^ and averaged for 128 scans at 4 cm^−1^ resolution. Data processing consisted of baseline smoothing using the automatic smooth processing function in the Spectrum software.

The frequency of the choline group (N(CH_3_)_3_^+^) asymmetric stretches was measured to the nearest 0.01 cm^−1^ from the peak absorbance of these bands, within approximately 970 cm^−1^ region [28]. The spectral changes with this vibrational have mode characteristic value, since it can provide valuable structural and conformational information about the changes occurring in the choline groups in the headgroup of the lipid molecules [28].

### 2.6. Hydrolysis of Phospholipid Molecules

Free fatty acid (FFA) content test kit (BC0695, Solarbio, Beijing, China) was used to detect FFA in hydrolysates during phospholipid storage. The principle of the test: FFA combines with copper ions to form fatty acid copper salts and dissolves in chloroform; the free fatty acid content can be deduced by measuring the copper ion content using the copper reagent method. Baseline calibration was re-prepared before each measurement.

### 2.7. GUVs Solution pH Measurement

The solutions of GUVs incubated at diverse temperatures (4 °C, 25 °C, 37 °C, 45 °C) for different times (0, 1, 3, 5, 7 days) were measured separately using a pH meter (S400, Mettler Toledo, Shanghai, China).

### 2.8. Date Analysis

Diameter measurements (>5.0 μm) of GUVs and aggregates (Contains 2, 3, 4, >4 GUVs), as well as number calculations, were performed in ImageJ version: 2.3 (ImageJ Software, Washington, DC, USA). Statistical analysis was performed using GraphPad Prism version 9.0 (GraphPad Software; San Diego, CA, USA) and Origin Lab version 8.5 (Origin Lab Software; Northampton, MA, USA) for Windows. All data are expressed as mean ± standard deviation (SD). Significant differences among groups were analyzed using a one-way ANOVA, and differences for individual groups were determined using Student’s *t*-test. The results were regarded as a significant difference when ** *p* < 0.01 and *** *p* < 0.001.

## 3. Results

### 3.1. Characterization of GUVs

**Figure 1:**
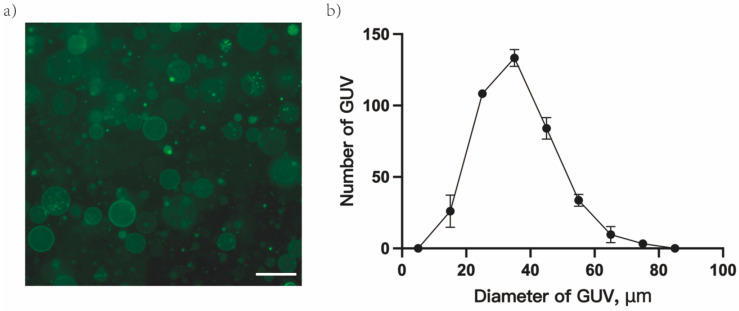
Basic properties of giant unilamellar vesicles(GUVs). (**a**) Light microscope observation of DPPC-GUVs (labeled by DiO) at RT. (**b**) Particle diameter distribution of GUVs; scale bar 100 μm.

Fresh GUVs’ morphology was observed via a light microscope, and their number and diameter distribution were measured to determine the filtering criteria for the GUVs solution. Under an optical microscope, DiO-labeled GUVs appeared round with good dispersion and no obvious aggregate (Figure 1a). Following statistical analysis, the number of GUVs in the field of view was in the range of 380–420, and the diameter distribution of GUVs displayed a normal distribution, with the bulk falling between 30 and 50 μm, and the diameter of most vesicles was approximately 37 μm (Figure 1b). GUV solutions satisfying the above screening criteria will be applied in subsequent experiments.

### 3.2. GUVs Aggregation by Increasing Temperature

**Figure 2:**
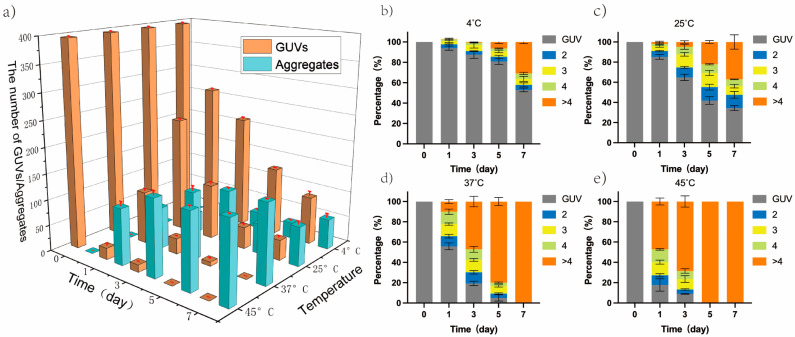
The degree of GUVs aggregation changed at different temperatures and incubation times. (**a**) Changes in the number of GUVs and aggregates from zero to seven days of incubation at 4 °C, 25 °C, 37 °C, and 45 °C; (**b**–**e**) Proportional variation of GUVs and different categories of aggregates (contained different number of GUV) at 4 °C, 25 °C, 37 °C, 45 °C from zero to seven days. Gray: GUV, blue: aggregates formed by two GUVs, Yellow: aggregates formed by three GUVs, Green: aggregates formed by four GUVs, Orange: aggregates formed by more than four GUVs.

To verify that temperature increases could promote the development of the GUVs from GUV to aggregate, the GUVs solutions were incubated at different temperatures. Additionally, two different metrics were applied in order to study the degree of aggregation of GUVs: the number of aggregates and the proportional distribution of different categories of aggregate. Under the light microscope, as temperature and duration of incubation increased, the degree of GUVs aggregation became increasingly apparent (Figure S1). Figure 2a confirms that, as temperature and incubation time rose, the number of GUVs gradually decreased from 390 (zero days) to 92 (4 °C, seven days), 39 (25 °C, seven days), 0 (37 °C, seven days; 45 °C, five days), and the number of aggregates gradually expanded to 58 (4 °C, seven days), 75 (25 °C, seven days), 150 (37 °C, seven days), and 154 (45 °C, seven days). Figure 2b–e reveals that the number of GUVs in the aggregates gradually increased with an increase in temperature and incubation time. The rate of aggregates (containing more than four GUVs) reached 100% after incubation at 37 °C for seven days and 45 °C for five days. Moreover, GUVs prepared from different phospholipids (DMPC, DPPC, DSPC) all showed the same tendency (Figure S2), with an increase in the percentage of the number of aggregates in solution with increasing temperature.

**Figure 3:**
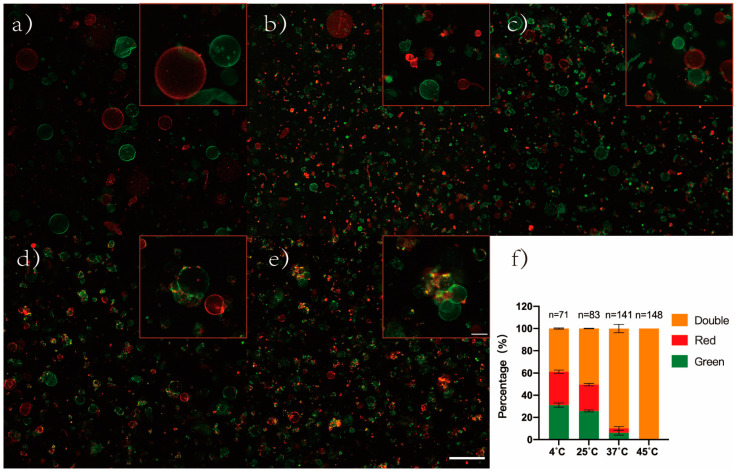
Incubation of DPPC-GUVs mixed solution (red GUV: labeled by DiI; green GUV: labeled by DiO). (**a**) Fresh GUVs; (**b**–**e**) dfGUVs were incubated at various temperatures for seven days (**b**: 4 °C; **c**: 25 °C; **d**: 37 °C; **e**: 45 °C); (**f**) The proportion of aggregates of different colors; scale bars 100 μm (**a**–**e** figures) and 20 μm (**a**–**e**: top right figures), respectively.

Next, the way of aggregate formation was explored. Different fluorescently labeled GUVs were prepared in the same way, including green GUVs (gGUVs, DiO labeled GUVs) and red GUVs (rGUVs, DiI labeled GUVs). Equal volumes of gGUVs and rGUVs solutions were mixed to generate GUVs mixed solution (mGUVs). The image of the mGUVs was captured using a CLSM, and the initial morphology of mGUVs was compared to that of mGUVs incubated at different temperatures for seven days (Figure 3a–e). GUVs were originally dispersed in the liquid, and no aggregation took place (Figure 3a). After seven days of incubation, the number of aggregates increased with increasing temperature, and pure color aggregates, as well as double-colored aggregates, could be observed under light microscopy (Figure 3b–e). In addition, no single GUV with both fluorescences was observed in the field of view

Figure 3f shows the classification of the aggregates, according to their colors, as well as the percentages of pure green fluorescence aggregate (containing only gGUVs), pure red fluorescence aggregate (containing only rGUVs), and double-color fluorescence aggregate (containing both gGUVs and rGUVs), which were all counted. The overall number of aggregates in the field of view increased with increasing temperature, reaching 71 (4 °C), 83 (25 °C), 141 (37 °C), and 148 (45 °C), respectively. The percentage of double fluorescent aggregates in the aggregates also increased, with percentages of 38.8% (4 °C), 50.4% (25 °C), 89.86% (37 °C), and 100% (45 °C), respectively. Then, an individual aggregate was directly visualized with the Z-axis layer sweep recombination technique by CLSM. The aggregate was shown from the XYZ-three-dimensional (consisting of 70 figures with a thickness of 0.5 μm per layer), XY-two-dimensional, and YZ-two-dimensional planes, respectively. Equal numbers of gGUVs and rGUVs are presented in the aggregates, which were formed by the tight contact of the GUV bilayers (Figure S3).

### 3.3. Effects of Increasing Temperature on GUVs Zeta Potential

**Figure 4:**
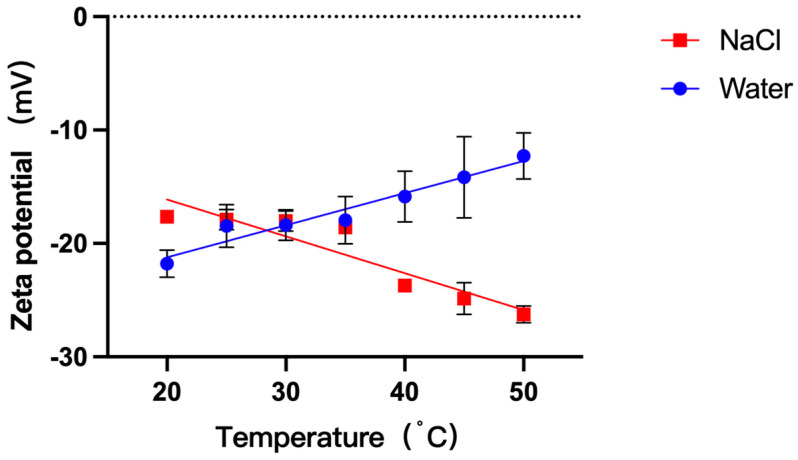
Zeta potential of DPPC-GUVs in water and 1 mM NaCl solution at different temperatures.

The zeta potential of GUVs was measured by optimized PALS (electric field strength: 11.11 V/cm) to reflect the electrostatic repulsive force between GUVs. The trends of the zeta potential of GUVs in 1 mM NaCl and in water were measured during increasing temperature. The zeta potential (absolute value) of GUVs in water gradually decreased with increasing temperature from −21.78 mV (20 °C) to −12.41 mV (50 °C). The zeta potential (absolute value) of GUVs in 1 mM NaCl solution gradually increased from −17.63 mV (20 °C) to −26.26 mV (50 °C). The zeta potential of GUVs in 1 mM NaCl showed an opposite trend to that in water (Figure 4).

### 3.4. Effect of Increasing Temperature on the Orientation of the Phospholipid Headgroup

**Figure 5:**
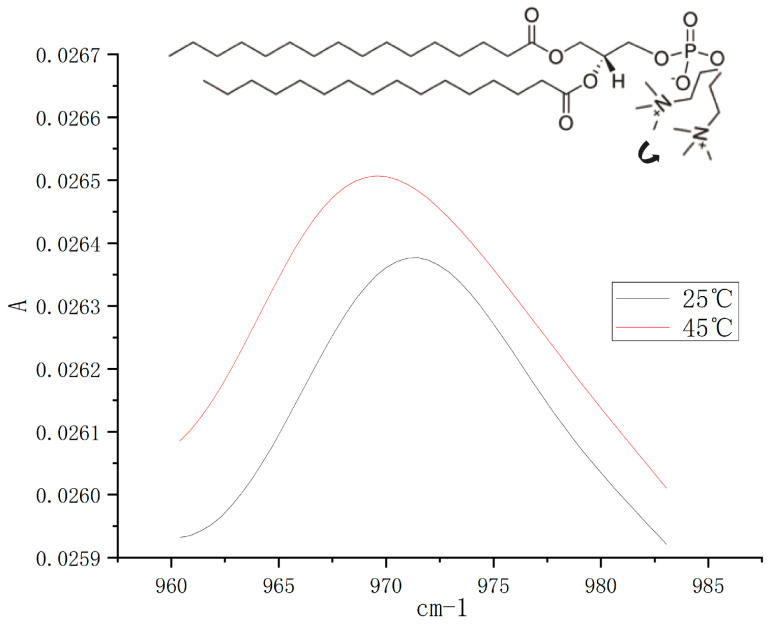
ATR-FTIR spectra of the choline groups of DPPC molecules at different temperatures. The inset indicates the directional change in the choline group after the temperature increase.

The change in vibrational frequency of the choline groups of the DPPC at different temperatures was measured using ATR-FTIR. The vibrational frequencies of the water were first examined, and the sample solutions of DPPC-MLVs at different temperatures were examined using the water as the background. As the temperature of the sample solution increased from 25 °C to 45 °C, the vibrational frequencies of the choline groups shifted to the left (Figure 5). Vibration frequency decreased from 971.49 ± 0.37 cm^−1^ to 969.84 ± 0.21 cm^−1^ (Table 1), and the data of the two groups were significantly different (*p* < 0.01). The decrease in the vibrational frequency of the choline group can usually reflect the tilt of the group toward the bilayer surface [29,30].

### 3.5. Effect of Zeta Potential on the Degree of GUVs Aggregation

**Figure 6:**
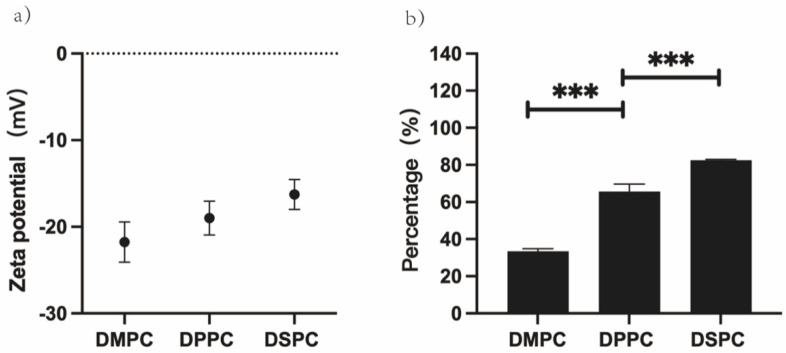
Zeta potential and degree of aggregation of GUVs prepared from DMPC, DPPC, and DSPC in water. (**a**) Differences in zeta potential in GUVs at 25 °C. (**b**) Differences in the degree of GUVs aggregation after seven days incubation at 25 °C; “***” indicate *p* < 0.001.

The relationship between zeta potential and the degree of aggregation of GUVs was further investigated. GUVs were prepared in water, utilizing different phospholipids (DMPC, DPPC and DSPC). The zeta potential of these GUVs was firstly measured, and the zeta potentials of GUVs were −21.76 mV (DMPC), −19 mV (DPPC), and −16.27 mV (DSPC), in that order (Figure 6a). Next, the aggregation of these GUVs at day 7 of incubation at 25 °C was compared, and the percentage of aggregates in the GUV solution was in the order of DMPC-GUVs (33.37%), DPPC-GUVs (65.57%), and DSPC-GUVs (82.5%), and the difference between the data was very significant (*p* < 0.001) (Figure 6b).

### 3.6. Effect of Ion on the Degree of GUVs Aggregation

**Figure 7:**
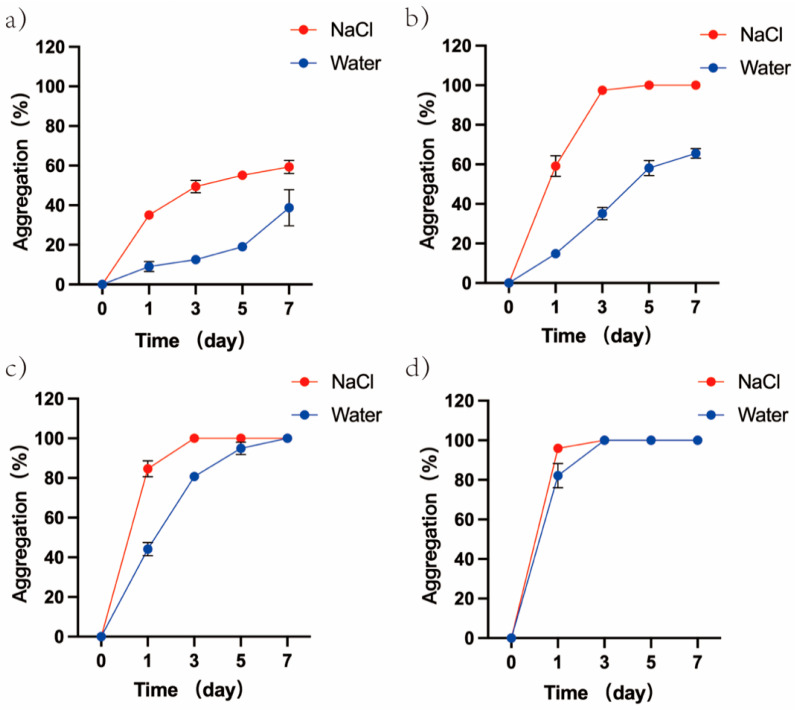
Differences in the percentage of aggregates between DPPC-GUVs incubated in 1 mM NaCl and water. (**a**) 4 °C; (**b**) 25 °C; (**c**) 37 °C; (**d**) 45 °C.

In the ionic solutions, the degree of aggregation of GUVs was observed during increasing temperature. As shown in Figure 7, the percentage of aggregates in both solutions expanded with rising temperature. On the 7th day of incubation at 4 °C, the percentage of aggregates in 1 mM NaCl solution reached 59.32%, while the percentage of aggregates in water reached only 38.77%. On day 5 of incubation at 25 °C, the percentage of aggregates in 1 mM NaCl reached 100%, while the percentage of aggregates in water reached only 58.13%. On day 3 of incubation at 37 °C, the percentage of aggregates in 1 mM NaCl reached 100%, while the percentage in water reached only 80.73%. On day 1 of incubation at 45 °C, the percentage of aggregates in 1 mM NaCl reached 95.99%, while the percentage of aggregates in water reached only 82.18%.

## 4. Discussion

The evolution of unicellular to multicellular is an important point in the evolution of life, and the emergence of multicellularity provides favorable conditions for the continuation and evolution of life [31]. Temperature, as an important and fundamental physical condition in the primitive environment, has a considerable impact on cell evolution [7,8]. At present, the effect of environmental temperature changes on the evolution of unicellular to multicellular evolution lacks attention, and the mechanism of its evolution remains to be studied. Therefore, it is essential to use cell models in the laboratory to study the effect of temperature changes on the evolution of unicellular to multicellular life. In this study, GUVs were prepared by electroformation using saturated phosphatidylcholine, which is the most abundant phospholipids in biological membranes [32]. The influence of temperature on the aggregation behavior of GUVs in cell models was investigated, and the potential mechanisms in this process were analyzed.

GUVs are frequently used as cell models and cell membrane models to investigate membrane behavior and function due to their similar size and membrane structure to cells [33]. In this study, the cell models were prepared in RNase-free water to remove the effect of proteins on their behavior [16]. The diameter distribution of the cell model prepared (5–85 μm) was analogous to that reported in the literature [34]. Under the microscope, the cell models were round and uniformly dispersed without aggregate. However, the accuracy of the subsequent experimental results will be impacted by the variations in the number and diameter distribution of models made in various batches [34]. Therefore, the screening criteria (Figure 1) for the diameter distribution of the GUVs solution were determined after several repeated experiments to exclude the interference of differences in size distribution and concentration in subsequent experiments.

Under the optical microscope, it was directly observed that, as temperature and incubation time increased, the number of aggregates in the visual field gradually increased, which was similar to the findings of Yin et al. [24], indicating that increasing temperature can promote vesicle aggregation. In order to reflect more objectively the relationship between temperature increase and the degree of aggregation of GUVs, the number of aggregates and the percentage of different categories of aggregates were counted under the microscope, respectively. The results showed that the number of aggregates in the field of view increased with the increase in temperature, and the number of GUVs in the aggregates also increased. The same trend was shown in the GUVs prepared from different phospholipids (DMPC, DPPC, DSPC) (Figure S2). It has been reported that the vesicles prepared by egg-PC, as well as non-phospholipid amphiles, are more prone to aggregation with increasing temperature [35,36,37,38]. This indicates a general promotion of vesicle aggregation by increasing temperature.

It has been reported that, in addition to the formation of aggregates by vesicle aggregation, the same phenomenon can be achieved by vesicle division [39]. By blending a solution of rGUVs and gGUVs in the same volume and incubating the mixed solution at different temperatures, the results showed that the number of aggregates increased with increasing temperature and that the largest proportion of aggregates was double-color fluorescent aggregates(containing both gGUVs and rGUVs). It indicates that GUV to aggregate occurs primarily through aggregation. The morphology of the aggregates observed under the microscope is similar to the cell colony model prepared by simple electrostatic attraction by Carrara et al. [40,41]. The cell models were tightly connected to each other.

Typically, the aggregation phenomenon of vesicle systems could be explained by the DLVO theory [42], which demonstrated that the van der Waals force and electrostatic repulsion cooperate to control vesicle aggregation. When the sum of the two forces is decreased to the same level as the thermal energy, each collision between vesicles will lead to aggregation [43]. It is believed that heating within a particular temperature range has little effect on van der Waals forces [44]. Therefore, vesicle aggregation may be the result of a decrease in electrostatic repulsion between vesicles.

The electrostatic repulsion between cell models can be reflected by measuring the zeta potential of the GUVs. However, Carvalho et al. pointed out that, when measuring the zeta potential of GUVs, excessive electric field strength may cause deformation of GUVs and make the measurement data inaccurate [45,46]. In this study, we first explored the effect of electric field strength of PALS on the zeta potential of GUVs (Figure S4) and the morphology of GUVs (Figure S5). The zeta potential of GUVs was measured at electric field strengths of 5.55 V/cm, 11.11 V/cm, and 22.22 V/cm, respectively, according to the electric field strength (12.5 V/cm) applied during the preparation of GUVs as the reference. The electric field strength 11.11 V/cm can better reflect the zeta potential of GUVs as a function of temperature compared to low electric field strength (5.55 V/cm). Compared with the high electric field strength (22.22 V/cm), the electric field strength 11.11 V/cm does not cause the deformation of GUVs, and the data error is small. The zeta potential of GUVs measured in all three electric field strengths, overall, exhibited a decrease in zeta potential with increasing temperature. However, the zeta potential trend of GUVs measured in the electric field intensity of 11.11 V/cm is more obvious, better reproducible, and has less effect on the morphology of GUVs.

Carvalho et al. made the first attempt to measure the zeta potential of GUVs [45], but also only to show the insertion of charged material into the bilayer and did not consider the effect of electric field strength on the morphology of GUVs [46,47]. To the best of our knowledge, there is no report on the measurement of the zeta potential of GUVs by optimizing the electric field strength. The results showed that the zeta potential of DPPC-GUVs (−19 mV) at 25 °C was close to that of DOPC-GUVs (−18 mV) reported in the literature [45], but greater than that of previous reports of DPPC-LUVs (−10 to 6 mV) [48,49,50], and the difference in zeta potential between the two vesicles may be related to molecular accumulation or vesicle curvature [48]. The zeta potential of DPPC-GUVs gradually decreases with increasing temperature (gradually approaching 0 mV). This is the same trend as that reported by Chibowski et al. [48], suggesting that increasing the temperature can decrease the zeta potential of vesicles.

Since the cell models used in this study were prepared from zwitterionic phospholipids, the zeta potential of zwitterionic phospholipid vesicles could reflect the phospholipid headgroup’s orientation with respect to the bilayer plane [23,51]. ATR-FTIR was used to detect the vibrational frequencies of the headgroups in DPPC in GUVs at different temperatures, but the concentrations were too low to discriminate the signals. As a result, instead of GUVs, high concentrations of DPPC-MLV suspensions were used for detection. The vibrational frequency of the choline group of DPPC was reported to be around 970 cm^−1^ [28], and the change in the vibrational frequency of the choline group could reflect the change in the direction of this group [29,30]. The results show that the vibrational frequencies of choline groups in DPPC decreased with increasing temperature (971.49 ± 0.37 cm^−1^ to 969.84 ± 0.21 cm^−1^), and Umemura et al. reported that the vibrational frequencies of choline groups in DOPC decreased with increasing temperature, following the same trend as in this paper [52]. As shown in Figure 5, increasing temperature caused the choline groups buried deep in the bilayer to tilt toward the bilayer plane, which is the same phenomenon reported by Doux et al. [53], indicating that increasing temperature can promote the tilt of choline groups in phospholipid molecules toward the bilayer.

In addition to the effect of temperature, the length of the hydrocarbon group chain influences the orientation of the headgroup of the phospholipid molecule [23]. The longer the hydrocarbon chain of the phospholipid, the closer the choline group in the phospholipid headgroup is to the bilayer plane [23]. As the choline group of DSPC was closer to the bilayer plane than that of DMPC, the zeta potential of DSPC-GUVs was smaller than that of DMPC-GUVs (Figure 6a), and the degree of aggregation of DSPC-GUVs was more obvious than DMPC-GUVs (Figure 6b). The zeta potential and degree of aggregation of DPPC-GUVs were intermediate between DMPC-GUVs and DSPC-GUVs. GUVs prepared from different phospholipids exhibit different zeta potentials due to the differences in the positions of choline groups in phospholipid molecules. The magnitude of the zeta potential can reflect the strength of electrostatic repulsion between GUVs. This is the reason why DSPC-GUVs are more likely to aggregate at 25 °C. It is undeniable that, at this temperature (25 °C), DMPC is in the liquid crystal phase, while DPPC and DSPC are both in the gel phase. The liquid crystal phase contributes to vesicle aggregation, but it is not entirely attributable to differences in the phase states.

It is well known that ions are involved in the evolution of life [54]. On the basis of the above, the effect of increasing temperature on cell models in ionic solutions was further investigated. The results showed that cell models in 1 mM NaCl solution are more easily aggregated than in aqueous solutions, and, as previously reported [14,55], this indicated that ions can promote the aggregation of vesicles.

The zeta potential of GUVs in ionic solutions was further examined. The zeta potential (absolute value) of GUVs in ionic solution gradually increased with increasing temperature, which was opposite to the trend of zeta potential change in GUVs in water. Knecht et al.’s simulation studies verified that Na^+^ and Cl^−^ ions have similar affinities for PC membranes [56]. However, for DPPC-GUVs presenting a weak negative potential, Na^+^ ions are more likely to bind to phosphate groups [55], resulting in a smaller measured absolute zeta potential of GUVs in ionic solution than in water (20–30 °C). Park et al. showed that low concentrations of ions can attenuate the zeta potential of DPPC vesicles [23,42,57]. Since low concentrations of NaCl (<10 mM) do not interfere with the motion of the choline group [53], progressively exposed choline groups attract more Cl^−^ to attach to the vesicle surface as the temperature increases, resulting in an enhanced zeta potential of the vesicle [53,58]. The electrostatic repulsive force between vesicles increased and hinders vesicle aggregation. It is difficult to state the difference in the aggregation rate of GUVs in 1 mM NaCl solution versus in water.

It is generally believed that, during incubation, phospholipid molecules are susceptible to hydrolysis and that the hydrolysis product fatty acids can promote vesicle aggregation [59,60]. The hydrolysis product fatty acids of phospholipid molecules were not detected in samples incubated at day 7 (Table S1), and Smistad et al. showed that the hydrolysis product was detected only after nine weeks of incubation at 35 °C [59]. The rate of hydrolysis of phospholipids is related to the pH of the solution and is lowest at pH values between 6.3 and 7.15 [61]. After testing, the pH of the GUVs solution was stable around 6.5 during the incubation period (Figure S6). This indicates that the aggregation phenomenon in this paper is not due to phospholipid hydrolysis and changes in the pH of the solution.

In addition to electrostatic repulsion, there may be other forces affecting the aggregation behavior of phospholipid vesicles, such as hydration repulsion [42]. With the increase in temperature, the dehydration capacity of polar headgroups of phospholipid molecules will gradually increase [24,25], leading to a decrease in the hydration repulsion between vesicles and promoting vesicle aggregation. In addition to the effect of temperature, ions in solution further disrupt the hydration repulsion between vesicles and promote vesicle aggregation [14,24]. Although the increasing electrostatic repulsion between GUVs in ionic solutions hinders the aggregation of GUVs as the temperature increases, the ions in solution can further disrupt the hydration repulsion between GUVs and promote the aggregation of GUVs. When the enhancement of electrostatic repulsive force cannot counteract the weakening of hydration repulsive force, the aggregation of vesicles can be promoted [14]. This is the main reason why vesicles are more likely to aggregate in ionic solutions [14].

The primitive Earth environment underwent multiple warming, as well as cooling [62], periods, and the changes in environmental temperature affect the evolution of life [7,8]. The evolution of the unicellular to the multicellular as an important point in the evolution of life brings many advantages to life. Firstly, multicellularity has a larger body size that facilitates cell survival [63,64,65,66]. Secondly a clearer division of labor within multicellularity helps the growth of the cell [64,67]. There are two academic views on how multicellular life evolved. One view is that single cells can evolve into multicellular life through cloning or self-division [65,67] The other view is that cells can achieve evolution to multicellularity by aggregation [68,69,70]. Although the mechanisms involved in the evolution of primitive single cells to multicellular life are still unclear, the studies in this paper suggest that changes in basic physicochemical conditions can promote aggregation in cell models. In the primitive environment, single cells can spontaneously aggregate into multicellularity. The aggregation of single cells can be effectively accelerated when the environmental temperature is increased or when ions are present in the solution.

## 5. Conclusions

In this paper, we investigate the effect of increasing temperature on the aggregation of cell models, and we explored the mechanism of this phenomenon. This study could effectively contribute to our understanding of the evolution of unicellular to multicellular life. In order to better adapt to environmental changes, primitive single cells can spontaneously aggregate into multicellularity. Changes in the basic physical and chemical conditions in the primitive environment can then accelerate the rate of single-cell aggregation. In fact, the evolutionary process of primitive unicellular to multicellular is more complex than we know and requires continuous in-depth study for better understanding of primal life.

## Figures and Tables

**Table 1:** Changes in the vibrational frequency of N(CH_3_)_3_^+^ region of DPPC molecules in different temperatures. “**” indicate *p* < 0.01.

Temperature	N(CH_3_)^3+^ Stretching (cm^−1^)
25 °C	971.49 ± 0.37 **
45 °C	969.84 ± 0.21 **

## Data Availability

Not applicable.

## Supplementary Materials

The following supporting information can be downloaded at: https://www.mdpi.com/article/10.3390/cimb45050242/s1, Figure S1: DiO-labeled GUVs morphology was observed under a microscope at different temperatures and times.; Figure S2: Degree of aggregation of GUVs prepared from DMPC, DPPC, and DSPC in water.; Figure S3: The aggregates were scanned with a laser confocal microscope to reconstruct the images (green GUV: labeled by DiO, red GUV: labeled by DiI) (70 × 0.5 μm); Figure S4: Zeta potential of DPPC-GUVs measured in various electric fields as a function of temperature; Figure S5: The morphology of DPPC-GUVs was observed under a microscope after different electric fields; Figure S6: pH changes in GUVs solution; Table S1: Free fatty acid content of GUVs suspensions after 7 days of incubated at different temperatures.
