# Supplementary material for: Temperature-Promoted Giant Unilamellar Vesicle (GUV) Aggregation: A Way of Multicellular Formation
Source: Curr Issues Mol Biol. 2023 Apr 26;45(5):3757–71. doi: 10.3390/cimb45050242 (PMC10217545; doi:10.3390/cimb45050242)
Supplement: Supplementary file 1 [file cimb-45-00242-s001.zip › cimb-2361666-supplementary.pdf]

Supplementary Materials:

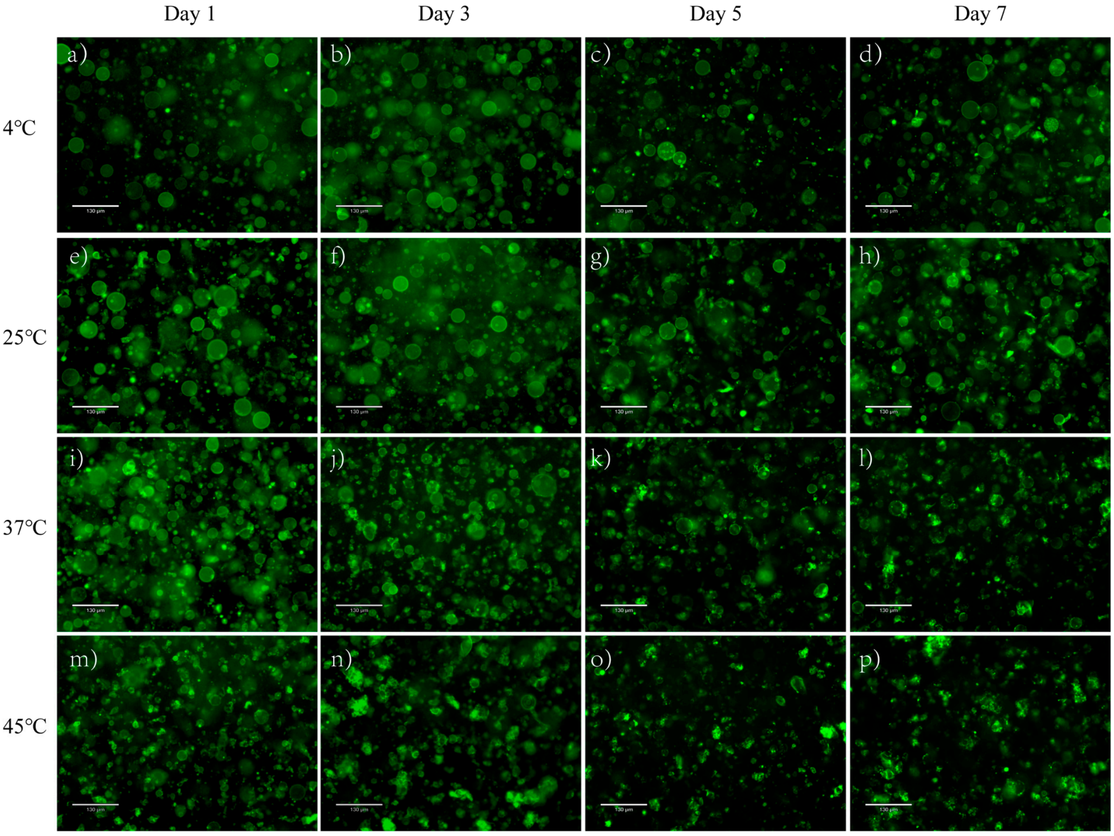

Figure S1. DiO-labeled GUVs morphology was observed under a microscope at different temperatures and times. a) 4˚C Day 1; **b)** 4˚C Day 3; **c)** 4˚C Day 5; **d)** 4˚C Day 7; **e)** 25˚C Day 1; **f)** 25˚C Day 3; **g)** 25˚C Day 5; **h)** 25˚C Day 7; **i)** 37˚C Day 1; **j)** 37˚C Day 3; **k)** 37˚C Day 5**; l)** 37˚C Day 7**; m)** 45˚C Day 1**; n)** 45˚C Day 3; **o)** 45˚C Day 5; **p)** 45˚C Day 7. scale bar 130 µm.

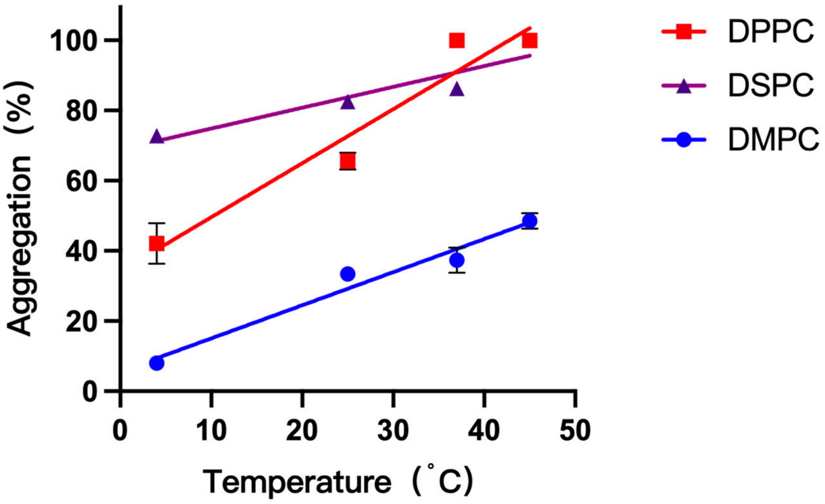

Figure S2. Degree of aggregation of GUVs prepared from DMPC, DPPC, and DSPC in water.

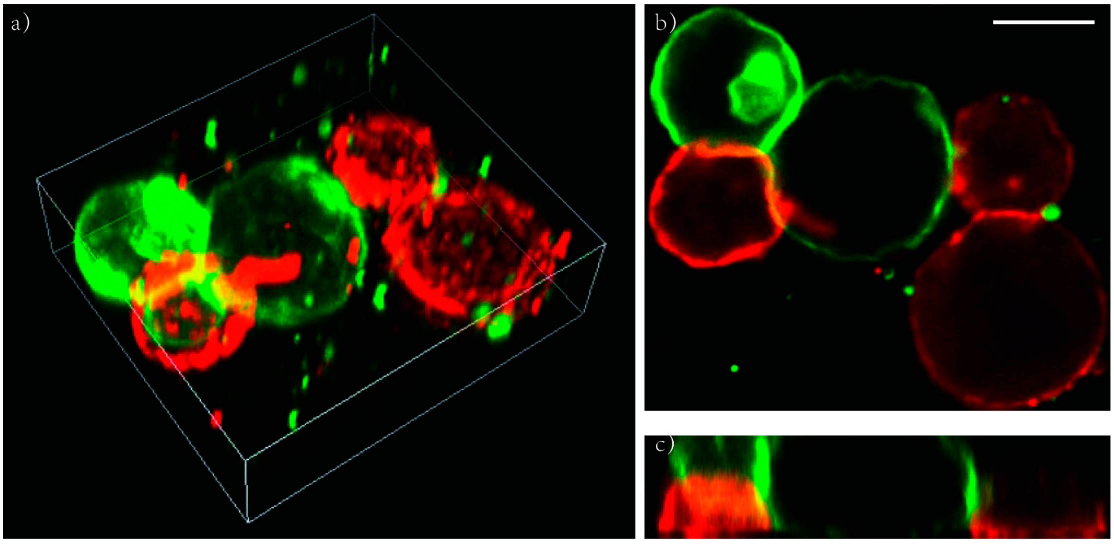

Figure S3. The aggregates were scanned with a laser confocal microscope to reconstruct the images (green GUV: labeled by DiO, red GUV: labeled by DiI) (70 × 0.5 µm). a) 3D-image; **b)** XY-2D-image; **c)** YZ-2D-image. scale bar 20 µm.

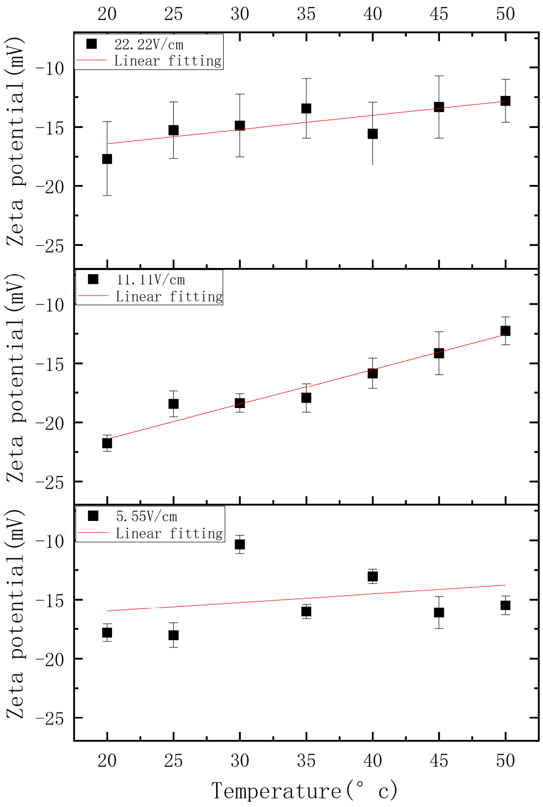

Figure S4. Zeta potential of DPPC-GUVs measured in various electric fields as a function of temperature.

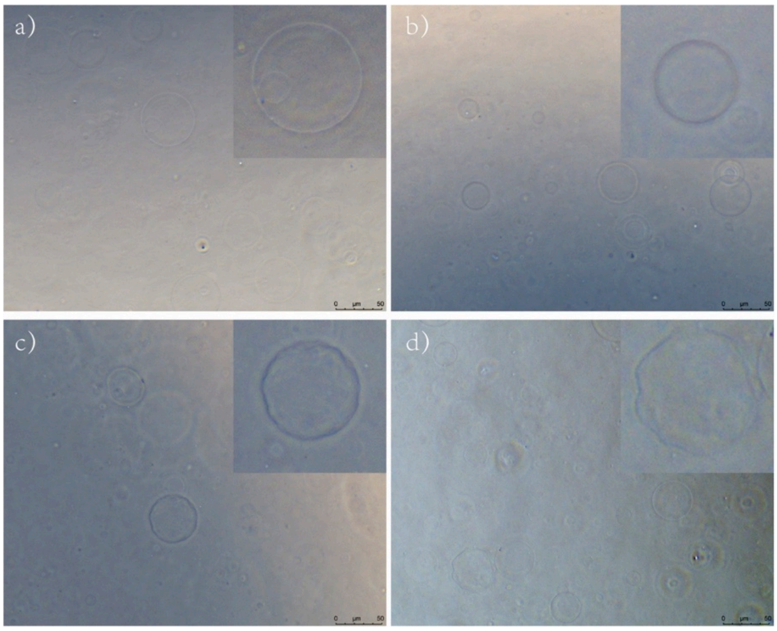

Figure S5. The morphology of DPPC-GUVs was observed under a microscope after different electric fields. a) 0 V/cm, **b)** 5.55 V/cm, **c)** 11.11 V/cm, **d)** 22.22 V/cm. scale bar 50 μm.

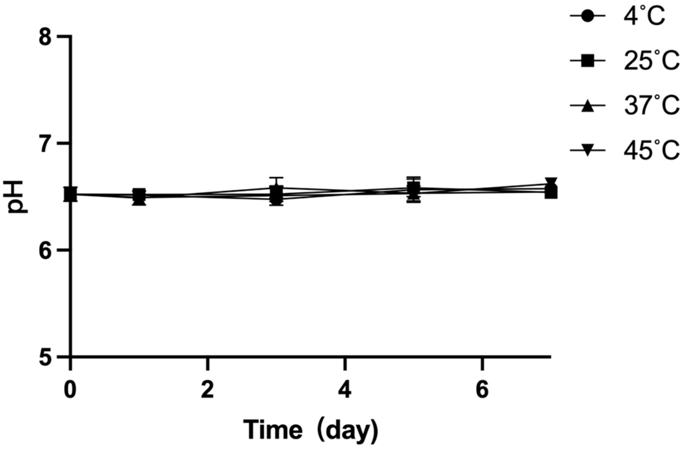

Figure S6. pH changes in GUVs solution.

Temperature	Free fatty acid concentration（μmol/mL）
4˚C (Day 7)	0
25˚C (Day 7)	0
37˚C (Day 7)	0
45˚C (Day 7)	0

Table S1 Free fatty acid content of GUVs suspensions after 7 days of incubated at different temperatures.
